# The sensitivity and significance analysis of parameters in the model of pH regulation on lactic acid production by *Lactobacillus bulgaricus*

**DOI:** 10.1186/1471-2105-15-S13-S5

**Published:** 2014-11-13

**Authors:** Ke Liu, Xiangmiao Zeng, Lei Qiao, Xisheng Li, Yubo Yang, Cuihong Dai, Aiju Hou, Dechang Xu

**Affiliations:** 1 School of Food Science and Engineering, Harbin Institute of Technology, Harbin, 150090, China

## Abstract

**Background:**

The excessive production of lactic acid by *L. bulgaricus *during yogurt storage is a phenomenon we are always tried to prevent. The methods used in industry either control the post-acidification inefficiently or kill the probiotics in yogurt. Genetic methods of changing the activity of one enzyme related to lactic acid metabolism make the bacteria short of energy to growth, although they are efficient ways in controlling lactic acid production.

**Results:**

A model of pH-induced promoter regulation on the production of lactic acid by *L. bulgaricus *was built. The modelled lactic acid metabolism without pH-induced promoter regulation fitted well with wild type *L. bulgaricus *(*R^2^_LAC _*= 0.943, *R^2^_LA _*= 0.942). Both the local sensitivity analysis and Sobol sensitivity analysis indicated parameters *T_max_, GR, K_LR_, S, V_0_, V_1 _*and *d_LR _*were sensitive. In order to guide the future biology experiments, three adjustable parameters, *K_LR_, V_0 _*and *V_1_*, were chosen for further simulations. *V_0 _*had little effect on lactic acid production if the pH-induced promoter could be well induced when pH decreased to its threshold. *K_LR _*and *V_1 _*both exhibited great influence on the producing of lactic acid.

**Conclusions:**

The proposed method of introducing a pH-induced promoter to regulate a repressor gene could restrain the synthesis of lactic acid if an appropriate strength of promoter and/or an appropriate strength of ribosome binding sequence (RBS) in *lacR *gene has been designed.

## Background

*Lactobacillus delbrueckii* subsp. *bulgaricus* has been widely applied in diary industry, especially as a starter for yogurt production. This microorganism is facultative anaerobic and can produce lactic acid. During the later period of fermentation and storage of yogurt, the production of lactic acid is dominated by *L. bulgaricus *[1]. The excessive production of lactic acid during storage makes the yogurt taste too sour and this phenomenon is called post-acidification.

At industrial level, several attempts have been made to slow down post-acidification, such as using devoid of *L. bulgaricus*, selecting the weak post-acidification *L. bulgaricus*, pasteurization after fermentation and so on [2-4]. However, these methods either control the post-acidification inefficiently or kill the probiotics in yogurt. Using genetic technology to modify the bacteria has become another option to decline post-acidification. Due to lacking molecular tools, mainly caused by the absence of a reliable transformation procedure, our understanding of the physiology and genetics of *L. bulgaricus *is still limited [5].

In *L. bulgaricus*, the absorption of lactose is processed by lactose/galactose antiport transport system [6]. Firstly, lactose is transported into cells by lactose permease encoded by *lacS *gene. Then the lactose is decomposed into glucose and galactose by β-galactosidase encoded by *lacZ *gene. The free galactose is pumped out of cells or stored in cells in the form of macromolecule i.e. carbohydrate gum. Glucose turns into pyruvate through glycolysis, and the glycolysis is transformed into lactic acid by the catalysis of lactate dehydrogenase [7-9]. The *lacS *gene and the *lacZ *gene constitute a *lacSZ *operon. In the downstream of *lacZ *gene, there is a *lacR *gene encoding a repressor which makes the *lacSZ *operon induced by lactose. However, in *L. bulgaricus*, the *lacR *gene has lost regulatory function due to the insertion of some gene fragments, resulting in constitutive expression of *lacSZ *operon [10].

Due to our limited knowledge, most attempts at the genetic level focused on changing the activity of one significant enzyme at a time in the metabolism of lactic acid. Druesne altered the Histidine codon to Alanine codon at the 552th locus of lactose permease, resulting in the reduction of the enzyme's activity [11]. Early in 1990, Mollet obtained the mutant *L. bulgaricus *without β-galactosidase activity by spontaneous deletion [12]. The new aforementioned organisms did produce less lactic acid but they had trouble in growing in milk independently due to the lack of energy. Adams selected two cold-sensitive mutants of the β-galactosidase from *L. bulgaricus *by using the expression system of *E. coli *[13]. However, their later research turned back to directly mutate and screen *L. bulgaricus *since it was difficult to transform with *L. bulgaricus*.

So far, *Lactobacillus delbrueckii* subsp. *bulgaricus *ATCC 11842 has been reported successfully transformed by electroporation, although with very low reproducibility and efficiency [5,14]. Several pH-induced promoters from *Lactococcus lactis *have been demonstrated, such as *rcfB *[15], P1 and P170 [16]. Combining the two points mentioned above, we came up with a new method to build a pH-induced promoter with a repressor gene controlling the production of lactic acid. Thus, we could turn on the switch at appropriate time. Here we analyse the parameters in the kinetic model to investigate the regulation effect of pH-induced promoter on lactic acid.

## Methods

### The reduced metabolic network

**Figure 1:**
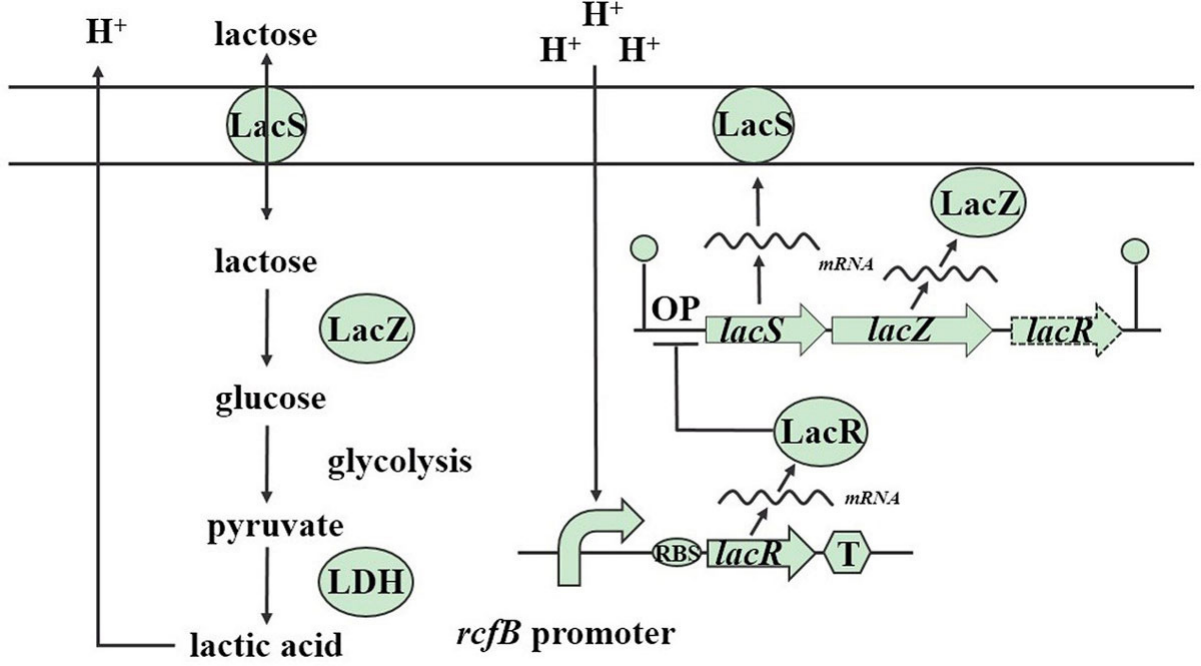
**A schematic of metabolic and gene regulation network model of the production of lactic acid**. The LacS, LacZ and LDH represent lactose permease, β-galactosidase, and lactate dehydrogenase respectively. When the extracellular pH reduces to 5.5, the pH-induced promoter (we chose *rcfB *promoter as an example) will trigger the express of LacR. The accumulation of LacR will combine with the operator sequence upstream of *lacS *gene, inhibiting the expression of LacS and LacZ [17].

Here we present a model of the production of lactic acid, which includes the important enzymes, lactose permease, β-galactosidase, lactate dehydrogenase and related regulation genes *lacS, lacZ, lacR*, as shown in Figure 1. The process of glycolysis is reflected in one reaction, adopting the assumption that there is a constant flux from glucose to pyruvate. Pyruvate is partly catalysed to lactic acid, and the other two products, acetyl-CoA and butanedione, are not taken into account.

### The mathematical model

The mathematical model describes how the extracellular pH value influences the production of lactic acid. The ordinary differential equations in the model are based on Michaelis-Menten equations [18] and fundamental kinetic principles. The variables are described in Table 1. The model assumptions are as follows.

**Table 1:** Variables used in the model

Variable	Description	Units
LAC_out_	Concentration of lactose out of the cells	mol/L
LAC_in_	Concentration of lactose in the cells	mol/L
GLC	Concentration of glucose in the cells	mol/L
PYR	Concentration of pyruvate in the cells	mol/L
LA	Concentration of lactic acid out of the cells	mol/L
LR, LS, LZ	Concentration of LacR protein, lactose permease, β-galactosidase in the cells	mol/L
M	Concentration of the mRNA of *lacR *gene	mol/L
N	Concentration of the mRNA of *lacS *and *lacZ *gene	mol/L
X	Concentration of cell mass	g/L

(1) Each entity, described in Table 1 is given as total amount in the population.

(2) The model does not take energy metabolism into consideration.

(3) All reactions are modelled by mass action principles, except for enzyme reactions which obey Michaelis-Menten equation, and transcription which obeys saturation kinetics.

(4) The level of translation product is assumed to be proportional to mRNA levels according to Dockery and Keener [19].

(5) There is no delay in synthesis of either component or delay because of protein transportations.

(6) All substances are subject to degradation.

#### The metabolism of lactose

As mentioned before, the metabolism of lactose is described by Michaelis-Menten equation. Since lactose transport is reversible, a term was included to count for lactose efflux dependent on the internal lactose concentration [20]. The *V_max _*for lactose permease and β-galactosidase is associated with the concentration of enzymes, so we use *K_cat _*and the concentration of enzymes to estimate the *V_max_*. Since the lac operon is usually induced by lactose, we assume that the repressor LacR protein could react with lactose. Thus we need to take the reduction into consideration when evaluating the concentration of intracellular lactose and LacR protein. As for the utilization of sugar, *L. bulgaricus *belongs to the type of homofermentation. Theoretically, this microorganism could totally convert glucose to lactic acid, but the conversion of glucose is about 80%-90% in practice [21]. Therefore, the estimated proportion of pyruvate transformed into lactic acid is 80%.

#### The gene regulation

The transcription and decomposition of lactose are tightly regulated at the genetic level: production of the enzymes can be decreased at the transcriptional level by regulatory protein LacR binding at appropriate DNA sites. We set M to stand for the transcript concentration of *lacR *gene. Since *lacS *and *lacZ *are located in the same operon, it is assumed that both genes are transcribed at same rate. We used N to describe the concentration of the two genes' mRNA. When describing the equations, the mRNAs are assumed to be transcribed at two distinct rates: basal one when there is no regulation and a higher/lower rate when being regulated [22].

#### The transcription of *lacR *gene

For M, it is described as function (1). The basal rate is given by *V_0 _*and increased at a constant rate *V_1 _*which is regulated by the pH value. *GR *stands for the repressor gene concentration. The degradation of the mRNA occurs at the rate of *d_M_*. It is also diluted due to cell growth rate at *μ*. We assumed the decrease of mRNA is first-order proportion to the concentration of M.

(1) $$\frac{dM}{dt}=\left(V_{0}+V_{1}\cdot F\left(pH\right)\right)\cdot GR-\left(d_{M}+\mu \right)\cdot M$$

As the mechanisms of pH-induced promoters have not been fully elucidated, we included a generic pH-dependent switch *F(pH) *which turns on when pH value is below the threshold. The switch takes the form of the smoothed step function [23], where 5.5 represents the threshold pH level around which the switch occurs [15]. *n *indicates the steepness of the smooth switch function.

(2) $$F\left(pH\right)=1-\text{tanh}\left(n\cdot \left(pH-5.5\right)\right)$$

Since lactic acid is the main product during fermentation, we assumed pH value is all controlled by the concentration of lactic acid. Besides, we needed to take the buffer capacity of milk medium into account. Furthermore, we assumed that all the lactic acid could spread to the medium even when the extracellular concentration was pretty high. We tested the pH value of milk medium when different amount of lactic acid was added. Three parallel experiments were taken and we fitted a function as the form of function (3). To the pH data, *c_0_, c_1 _*and *c_2 _*are constants. This function was then fed into the model via the switch function *F(pH)*.

(3) $$pH=c_{0}-c_{1}\cdot LA+c_{2}\cdot {\left(LA\right)}^{2}$$

#### Transcription of *lacS *and *lacZ *gene

For N, it is described as function (4). The basal rate is given by *V_2 _*and the transcription is inhibited by LacR. For the part to simulate the regulation rate of LacR, *T_max _*stands for the maximal rate of *lacS *and *lacZ *gene transcription, *S *represents the sensitivity of *lacS *and *lacZ *gene transcription to lactose permease and β-galactosidase [24]. *G *is the concentration of transcription gene. Again, we assumed first-order degradation rate at *d_N _*and dilution rate at *μ *of the mRNA.

(4) $$\frac{dN}{dt}=\left(V_{2}-\frac{T_{\text{max}}\cdot S\cdot LR}{1+S\cdot LR}\right)\cdot G-\left(d_{N}+\mu \right)\cdot N$$

#### Cell growth

In milk medium, lactose is the main carbon source, so it is assumed that the cell growth rate is dependent on the extracellular lactose concentration. The product, lactic acid, which declines the pH value of milk medium performs inhibition on cell growth. The function to describe the production inhibition is the same as Concepcion presented [21]. In function (5), *K_s _*is the Monod constant for growth in extracellular lactose, and *μ_max _*is the maximum specific growth rate. *K_LA _*is the maximum initial lactic acid concentration in which the microorganism growth is completely inhibited.

(5) $$\mu =\mu_{\text{max}}\cdot \left(\frac{LAC_{out}}{LAC_{out}+K_{s}}\right)\cdot \left(1-\frac{LA}{K_{LA}}\right)$$

#### Combining the metabolism of lactose with gene regulation

All the definitions of the parameters are shown in Table 2. Combining with the enzyme reactions, gene regulations and cell growth discussed above, the process described in Figure 1 could be represented by:

**Table 2:** List of parameters used in the model

parameter	definition	value	reference/derivation
*K_m·LP_*	Michaelis constant for lactose permease	2.6 × 10^-4 ^M	Patrick *et al*. (1997) [20]
*K_cat·LP_*	rate constant for lactose transportation	1.29 × 10^5^h^-1^	Patrick *et al*. (1997)
*K_m·GAL_*	Michaelis constant for β-galactosidase	9.8 × 10^-4^M	Moez *et al*. (2009) [25]
*K_cat·GAL_*	hydrolysis rate for lactose	1.65 × 10^5^h^-1^	Moez *et al*. (2009)
*K_m·GLYC_*	Michaelis constant for glycolysis	1 × 10^-4^M	*L. Lactis *Marcel *et al*. (2002) [26]
*V_max·GLYC_*	maximum reaction speed when enzymes was saturated by substrate	143.82M·h^-1^	*L. Lactis *Marcel *et al*. (2002)
*K_m·LDH_*	Michaelis constant for lactate dehydrogenase	0.1 M	*L. Lactis *Marcel *et al*. (2002)
*V_max·LDH_*	maximum reaction speed when lactate dehydrogenase was saturated by pyruvate	307.08M·h^-1^	*L. Lactis *Marcel *et al*. (2002)
*K_LS_*	LacS synthesis rate constant	564 h^-1^	Kennell *et al*. (1977) [27]
*d_LS_*	LacS decay rate constant	0.6 h^-1^	Kennell *et al*. (1977)
*K_LZ_*	LacZ synthesis rate constant	1.13 × 10^3^h^-1^	Kennell *et al*. (1977)
*d_LZ_*	LacZ decay rate constant	0.6 h^-1^	Kennell *et al*. (1977)
*K_LR_*	LacR synthesis rate constant	564 h^-1^	Assumed
*d_LR_*	LacR decay rate constant	0.6 h^-1^	Assumed
*d_M _, d_N_*	mRNA decay rate constant	41.58 h^-1^	Varmus *et al*. (1970) [28] and Patrick *et. al*. (1997)
*GR, G*	*lacR, lacSZ *gene concentration	2.5 × 10^-9 ^M	Cheng *et al*. (2001) [29]
*n*	steepness of the smooth switch function	1	Akyol *et al*. (2008) [15]
*K_b_*	LacR/lactose binding rate constant	7.2 × 10^4 ^M^-1^·h^-1^	Assumed
*V_0_*	basal rate of *lacR *gene transcription for mRNA	1 h^-1^	Assumed
*V_1_*	increasing constant of *lacR *gene transcription for mRNA	20 h^-1^	Assumed
*V_2_*	basal rate of *lacSZ *gene transcription for mRNA	3.5 h^-1^	Obtained by 'fitting'
*T_max_*	maximum rate of *lacSZ *gene transcription	6 × 10^4 ^h^-1^	Assumed
*S*	sensitivity of *lacSZ *gene transcription to LacS and LacZ	1000	Guessed
*K_S_*	saturation constant for growth of *L. bulgaricus *on extracellular lactose	9.82 × 10^-3 ^M	Concepcion *et al*. (2000) [21]
*K_LA_*	maximum lactic acid concentration to inhibit the growth of *L. bulgaricus*	0.448 M	Concepcion *et al*. (2000)
*μ*	maximum growth rate	0.8 h^-1^	Concepcion *et al*. (2000)

(6) $$\begin{array}{c} R_{1}=\frac{K_{cat\cdot LP}\cdot LS\cdot LAC_{out}}{LAC_{out}+K_{m\cdot LP}}\\ R_{2}=\frac{K_{cat\cdot LP}\cdot LS\cdot LAC_{in}}{LAC_{in}+K_{m\cdot LP}}\\ R_{3}=\frac{K_{cat\cdot GAL}\cdot LZ\cdot LAC_{in}}{LAC_{in}+K_{m\cdot GAL}}\\ R_{4}=\frac{V_{\text{max}\cdot GLYC}\cdot GLU}{GLU+K_{m\cdot GLYC}}\\ R_{5}=\frac{V_{\text{max}\cdot LDH}\cdot PYR}{PYR+K_{m\cdot LDH}}\\ R_{6}=V_{0}\cdot GR\\ R_{7}=V_{1}\cdot F\left(pH\right)\cdot GR\\ R_{8}=\left(d_{M}+\mu \right)\cdot M\\ R_{9}=K_{LR}\cdot M\\ R_{10}=K_{b}\cdot LR\cdot LAC_{in}\\ R_{11}=\left(d_{LR}+\mu \right)\cdot LR\\ R_{12}=V_{2}\cdot G\\ R_{13}=\left(\frac{T_{\text{max}}\cdot S\cdot LR}{1+S\cdot LR}\right)\cdot G\\ R_{14}=\left(d_{N}+\mu \right)\cdot N\\ R_{15}=K_{LS}\cdot N\\ R_{16}=\left(d_{LS}+\mu \right)\cdot LS\\ R_{17}=K_{LZ}\cdot N\\ R_{18}=\left(d_{LZ}+\mu \right)\cdot LZ\\ R_{19}=\mu \cdot X \end{array}$$

The final metabolic model is given by:

(7) $$\begin{array}{c} \frac{dLAC_{out}}{dt}=-R_{1}+R_{2}\\ \frac{dLAC_{in}}{dt}=R_{1}-R_{2}-R_{3}-R_{10}\\ \frac{dGLU}{dt}=R_{3}-R_{4}\\ \frac{dPYR}{dt}=2R_{4}-0.8R_{5}\\ \frac{dLA}{dt}=0.8R_{5}\\ \frac{dM}{dt}=R_{6}+R_{7}-R_{8}\\ \frac{dLR}{dt}=R_{9}-R_{10}-R_{11}\\ \frac{dN}{dt}=R_{12}-R_{13}-R_{14}\\ \frac{dLS}{dt}=R_{15}-R_{16}\\ \frac{dLZ}{dt}=R_{17}-R_{18}\\ \frac{dX}{dt}=R_{19} \end{array}$$

We set stoichiometric constant of *R_4 _*with two since two molecules of pyruvate are formed from one molecule of glucose. For the stoichiometric constant of *R_5_*, it stands for the proportion of pyruvate which is converted to lactic acid.

#### Data estimated

Firstly, we did not take *lacR *gene into consideration. This is just the case of wild type *L. bulgaricus *which *lacSZ *operon is constitutive expression. Since most of the parameters in this reduced model have been reported, *V_2 _*is the only one needs to be modified. We used SBToolbox in Matlab to set up the reduced model and simulated the metabolism process [30]. We compared the concentration changes of extracellular lactose and lactic acid within 8 hours with the data from Fatama, where the conditions used were 43 °C, 8% of skim milk concentration (without pH value control) and 4% of inoculum ratio [31]. After that, we added *lacR *gene. This time we did not have sufficient data for full parameterization of the model. However, we did have enough information to estimate their relative sizes and give the qualitative nature of our investigations. The parameter values are listed in Table 2.

### Sensitivity and significance analysis of parameters

We still used SBToolbox to perform local sensitivity analysis and Sobol's method for global sensitivity analysis. The local sensitivity analysis investigates how small changes in a single parameter value could affect the model output. The method is based on the partial differentiation of the output with respect to the input parameters [32]. Herein, the partial differentiation is evaluated numerically by introducing a 1% increment from the specific parameter value. We chose Sobol sensitivity analysis method to calculate global sensitivity since the sensitivity index quantified the overall effects of a parameter, in combination with any other parameters, on the model output [33]. The number of simulation to carry out is 10000 times. We first set the number as 1000 according to Schmidt [30]. Since the results are varied among different simulations, we increased the number to 10000 and got stable outcomes. During the above sensitivity analysis, we chose lactic acid concentration as the model output.

After figuring out the sensitivity parameters to the model output, we carried out different simulations with each sensitivity parameter at two levels, a relative low value and a relative high value. The metabolism of lactic acid and changing of pH values was carefully considered. Those results indicate how pH values affect lactic acid production under different situations and help us to understand how to design LacR regulation in biology experiments in future.

## Results and discussion

### Reduced model without *lacR *gene

**Figure 2:**
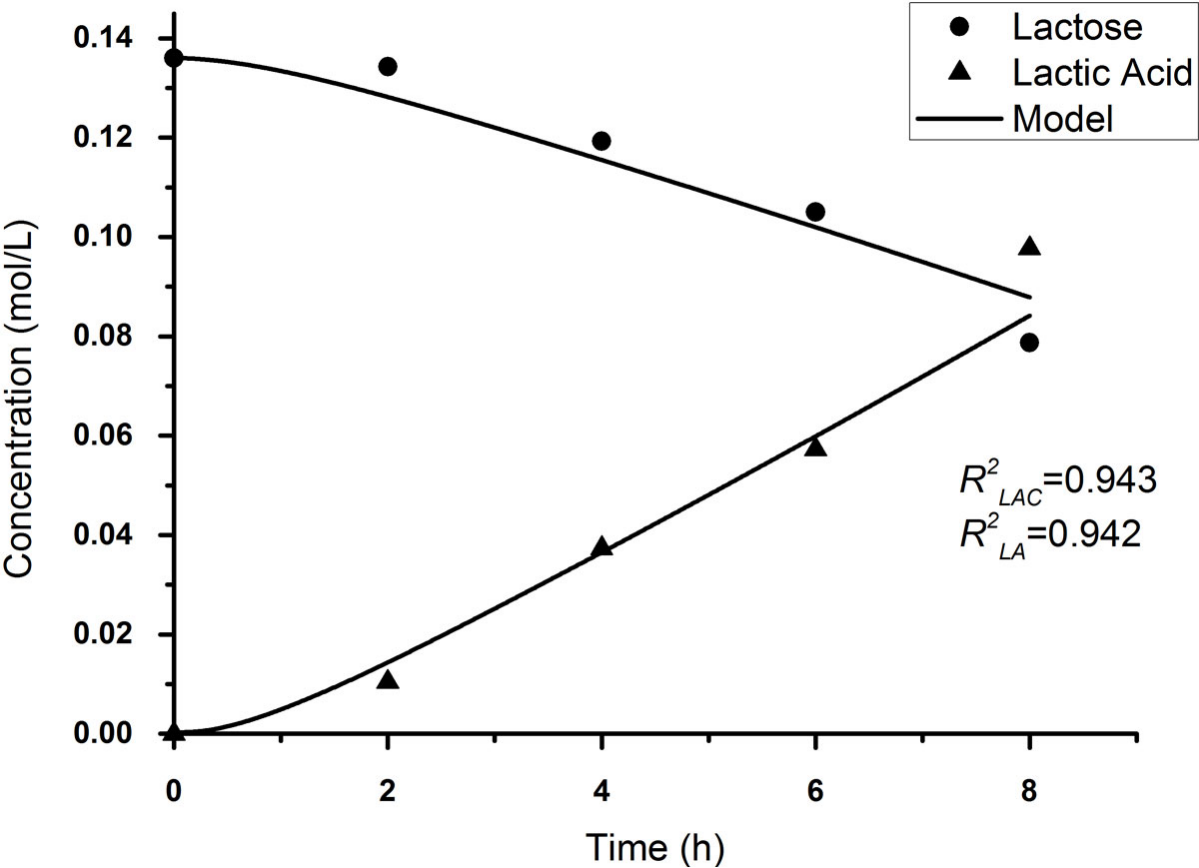
**Simulation of batch fermentation conversion of lactose to lactic acid using wild type *L. bulgaricus***. The initial conditions were 43 °C 8% of skim milk concentration (without pH value control) and 4% of inoculum ratio. The dots and triangles indicated the measured concentration of lactose and lactic acid in the medium, respectively, every two hours. The curves were the simulated results of lactose and lactic acid concentration in 8 hours.

The results of modelled concentration changing of extracellular lactose and lactic acid are shown in Figure 2, along with the experimental data. The model fits the experimental data well. We have obtained the value of *V_2_*, which indicates the basal rate of *lacSZ *gene transcription. The relative size of fitting value (*V_2 _*= 3.5 h^-1^) is determined according to Gustafsson's [24] report.

### Sensitivity analysis of parameters

**Figure 3:**
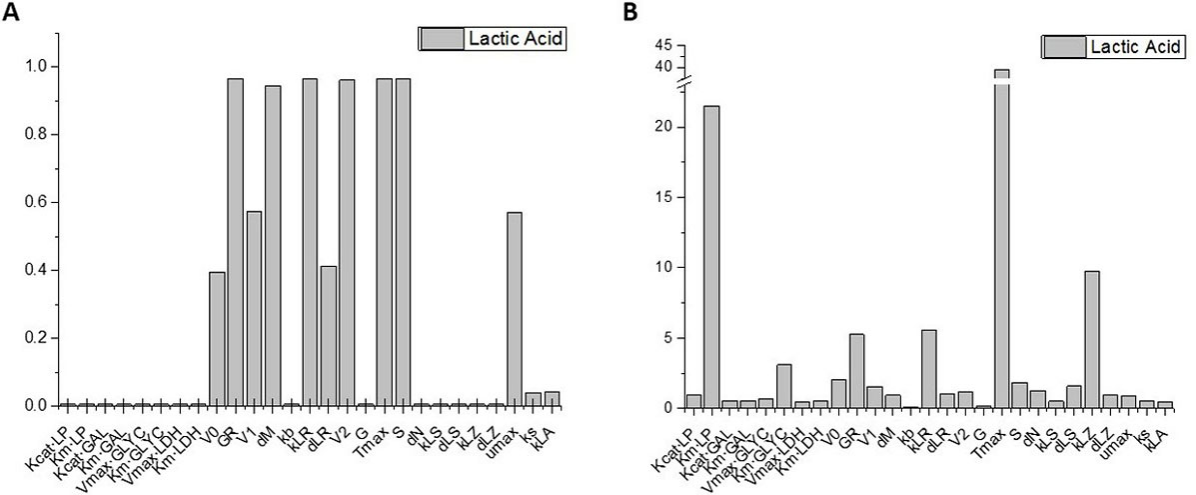
**Local sensitivity analysis and Sobol's method of global sensitivity analysis**. A Local sensitivity analysis B Sobol's method of global sensitivity analysis

The results of local sensitivity analysis and Sobol sensitivity analysis are shown in Figure 3. The parameters with sensitivity from high to low in local sensitivity analysis are followed as *T_max_, GR, K_LR_, S, V_2_, d_M_, V_1_, u_max_, d_LR _*and *V_0_*. The other parameters perform little effect on the production of lactic acid. The reason is that when one of those parameters increased, it would only affect the production rate of the output but have no effect on the total amount of lactic acid. Thus, they are not sensitive parameters in this method. As for Sobol sensitivity analysis, the parameters with sensitivity from high to low is *T_max_, K_m·LP_, K_LZ_, K_LR_, GR, K_m·LYC_, V_0_, S, d_LS_, V_1_, d_N_, V_2 _*and *d_LR_*. The parameter *K_m·LP _*shows the second most sensitivity in Sobol's method, just as Druesne has proven, the reduction of lactose permease's activity would greatly decline the production of lactic acid [11].

### Significant analysis of parameters

In sensitivity analysis, sensitivity does not mean importance, since sensitive parameters are always fixed inherently and unadjustable. We need to combine the sensitivity results with operability, especially for the design of biology experiments. Although *T_max_, K_LZ_, V_2_, d_LS_, d_LR_, GR, d_M_, d_N _*and *u_max _*perform high sensitivity in both methods, these parameters are usually inherently. *K_m·LP _*value is adjustable, but to decrease its value would generate fatal disadvantage on the growth of the bacteria. So we do not include this parameter into consideration. Due to the same reason, we exclude *K_m·GLYC_*, which is the Michaelis constant for glycolysis. As for the parameter *S*, which means sensitivity of *lacSZ *gene transcription to LacS and LacZ proteins, it is difficult to measure and adjust in experiments. Therefore, the parameters which exhibit great influence on the production of lactic acid in both methods are *K_LR_*, *V_0 _*and *V_1_*, with *K_LR _*representing RBS strength of *lacR *gene, and *V_0 _*together with *V_1 _*reflecting characters of the pH-induced promoter.

**Figure 4:**
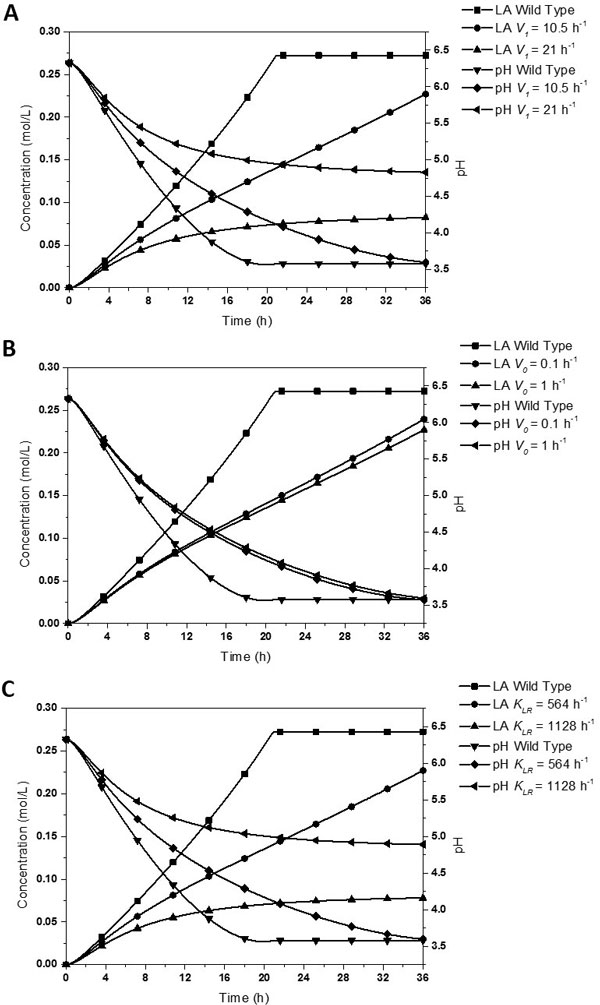
**The effects of *V_1_, V_0 _*and *K_LR _*on the production of lactic acid and pH**. The variations of lactic acid and pH in wild type are set as blank control. The curves indicated the simulated concentration of lactose and pH along with time under different values of *V_1_, V_0 _*and *K_LR_*. A The effects of *V_1 _*(10.5 h^-1 ^or 21 h^-1^), where *V_0 _*= 1 h^-1^, *K_LR _*= 564 h^-1^. B The effects of *V_0 _*(0.1 h^-1 ^or 1 h^-1^), where *V_1 _*= 10.5 h^-1^, *K_LR _*= 564 h^-1^. C The effects of *K_LR _*(564 h^-1 ^or 1128 h^-1^), where *V_0 _*= 1 h^-1^, *V_1 _*= 10.5 h^-1^

For the three parameters, the relative high levels of *K_LR_, V_0 _*and *V_1 _*are 1128 h^-1^, 1 h^-1 ^and 21 h^-1 ^respectively. The relative low levels of *K_LR_, V_0 _*and *V_1 _*are 564 h^-1^, 0.1 h^-1^, 10.5 h^-1 ^respectively. The results are shown in Figure 4. From picture A, we could figure out that with the increase of promoter strength, the production rate and amount of lactic acid would be greatly decreased. However, for the relative high value of *V_1_*, the finial pH value only declines to 4.83 which is higher than casein isoelectric point. Thus we need to find an appropriate promoter strength between the relative high and low values when it is turned on by pH value. From picture B, we could draw the conclusion that if the pH-induced promoter could be efficiently turned on when pH decreases to its threshold, leaking expression of the promoter would have little effect on the production of lactic acid. In picture C, the increase of *K_LR _*leads to strong decline of both the production rate and total amount of lactic acid. By comparing the results of relative high level *K_LR _*with the relative high level *V_1 _*in picture A, the two parameters almost perform the same influence on the production of lactic acid. This is due to the reason that the two parameters both affect the concentration of LacR. Therefore, we could adjust the strength of pH-induced promoter and/or RBS strength of *lacR *gene to make our switch perform well in controlling lactic acid production.

Other two important features for the promoter are the threshold pH level around which it would start and whether the promoter would keep turning on when the pH value decreased to 3.5 or lower. Akyol [15] and Madsen [16] only described the status of pH-induced promoters along with the pH declined to 5.5. Further experiments are needed to test the characteristics of the promoters. This model neglect the effect of lower pH on the promoter, assuming that it would still turn on with the decrease of pH value.

## Conclusions

In conclusion, we propose a new method to control the production of lactic acid by *L. bulgaricus *which is to build a pH-induced switch (promoter) to turn on repressor gene. The proposed method overcomes the disadvantage in bacterial growth by directly changing one of the enzyme related to lactic acid metabolism. Then, we build a reduced model of pH-induced promoter regulation on the production of lactic acid and adjust the model with data from wild type *L. bulgaricus*. After that we carry out sensitivity analysis of the parameters and figure out three significant ones in the model. To make our switch work well, we need to find an appropriate strength of promoter and/or an appropriate strength of RBS in *lacR *gene. The values of those two parameters should between the high level and low level we have set in the analysis, so that when the pH value declines to the promoter's starting point, the *lacR *gene could express moderately to inhibit the production of lactic acid.

## Competing interests

The authors declare that they have no competing interests.

## Authors' contributions

KL, LQ, XL, YY proposed the project and designed metabolic and gene regulation network. KL and XZ wrote the model and performed sensitivity analysis. CD, AH and DX guided the model network design and significance analysis of the parameters. KL wrote the manuscript. All authors have read and approved the final manuscript.

## Declarations

This work and its publication is partially supported by the China Natural Science Foundation (Grant Number: 30771371, 31271781), the National High-tech R&D Program of China (863 Program) (Grant Number: 2001AA231091, 2004AA231071), Heilongjiang Province Science Foundation (Grant Number: 2004C0314), Heilongjiang Province key scientific and technological project (Grant Number: WB07C02), HIT Science Foundation (Grant Number: HIT. 2003. 38), National MOST special fund (Grant Number: KCSTE- 2000-JKZX- 021, NCSTE- 2007- JKZX- 022, 2012EG111228).

This article has been published as part of *BMC Bioinformatics *Volume 15 Supplement 13, 2014: Selected articles from the 9th International Symposium on Bioinformatics Research and Applications (ISBRA'13): Bioinformatics. The full contents of the supplement are available online at http://www.biomedcentral.com/bmcbioinformatics/supplements/15/S13.
